# Resource-sharing between internal maintenance and external selection modulates attentional capture by working memory content

**DOI:** 10.3389/fnhum.2014.00670

**Published:** 2014-08-27

**Authors:** Anastasia Kiyonaga, Tobias Egner

**Affiliations:** Department of Psychology and Neuroscience and Center for Cognitive Neuroscience, Duke UniversityDurham, NC, USA

**Keywords:** working memory, attention, visual search, resource-sharing

## Abstract

It is unclear why and under what circumstances working memory (WM) and attention interact. Here, we apply the logic of the time-based resource-sharing (TBRS) model of WM (e.g., Barrouillet et al., 2004) to explore the mixed findings of a separate, but related, literature that studies the guidance of visual attention by WM contents. Specifically, we hypothesize that the linkage between WM representations and visual attention is governed by a time-shared cognitive resource that alternately refreshes internal (WM) and selects external (visual attention) information. If this were the case, WM content should guide visual attention (involuntarily), but only when there is time for it to be refreshed in an internal focus of attention. To provide an initial test for this hypothesis, we examined whether the amount of unoccupied time during a WM delay could impact the magnitude of attentional capture by WM contents. Participants were presented with a series of visual search trials while they maintained a WM cue for a delayed-recognition test. WM cues could coincide with the search target, a distracter, or neither. We varied both the number of searches to be performed, and the amount of available time to perform them. Slowing of visual search by a WM matching distracter—and facilitation by a matching target—were curtailed when the delay was filled with fast-paced (refreshing-preventing) search trials, as was subsequent memory probe accuracy. WM content may, therefore, only capture visual attention when it can be refreshed, suggesting that internal (WM) and external attention demands reciprocally impact one another because they share a limited resource. The TBRS rationale can thus be applied in a novel context to explain why WM contents capture attention, and under what conditions that effect should be observed.

## Working memory (sometimes) biases visual attention

Working memory (WM) typically describes the short-term maintenance and manipulation of internal information (i.e., no longer available to the senses), but the material being maintained in WM can impact the focusing of attention toward external stimuli. WM can be volitionally used to maintain current goals and guide where attention is allocated (Bundesen, 1990; Desimone and Duncan, 1995; Woodman and Chun, 2006), but items matching WM content can also involuntarily capture visual attention in an unrelated task (e.g., Soto et al., 2005; Olivers et al., 2006). The current study explores boundary conditions of this WM biasing, and bridges two separate literatures to test a mechanism that might explain how WM and attention are linked.

A high load on WM storage can impair performance of an attention-demanding task (e.g., De Fockert, 2001; Chen and Cowan, 2009), and items maintained in WM can guide eye movements and attention toward matching, but task-irrelevant, items when a visual search occurs during the WM delay interval (for reviews see Soto et al., 2008; Olivers et al., 2011). Visual search is typically speeded, for instance, if a memory-matching item coincides with a search target (a valid trial), and slowed if a memory-matching item coincides with a search distracter (Soto et al., 2005). Attention can be captured by external WM-matching items even when the WM content never cues the search target (Soto et al., 2005; Olivers et al., 2006), and no memory probe is given after the search array (Kiyonaga et al., 2012)—conditions under which there would be no incentive to voluntarily attend to the WM-match. Moreover, WM content can capture attention above and beyond highly perceptually salient and “pop-out” targets (Soto et al., 2006; Dowd and Mitroff, 2013), and even in a simple detection task with no competition for selection among stimuli (Hollingworth et al., 2013), suggesting that the link between WM representations and visual orienting is obligatory and has its impact early in the processing stream. These observations are consistent with the biased competition model of attention (Desimone and Duncan, 1995), wherein the active representation of an item being held in WM causes stimuli in the environment matching those maintained features to be preferentially attended (i.e., win the competition for selection).

There are also many instances, however, when WM and attention processes can occur simultaneously without impeding one another (e.g., we are generally able to rehearse our to-do list while also operating a car and following traffic signals). Accordingly, in some studies attention task performance has remained efficient despite concurrent WM storage demands (Woodman et al., 2001; Cocchini et al., 2002), and WM content has failed to involuntarily capture visual attention (Downing and Dodds, 2004; Houtkamp and Roelfsema, 2006; Woodman and Luck, 2007; Peters et al., 2009). Why might WM and attention demands impact one another in some situations but not others? These inconsistencies fuel an ongoing debate about the degree of overlap between the content and function of WM and attention, and experimental attempts to explain the conflicting results (Soto and Humphreys, 2008; Han and Kim, 2009; Olivers, 2009; Dombrowe et al., 2010; Zhang et al., 2010, 2011; Tsvetanov et al., 2012) have yet to establish a clear set of rules governing how and when WM will impact attention—and specifically when WM content will capture visual attention in an unrelated task. We postulate that the framework of a fruitful model of the relationship between WM storage and processing can be applied to potentially resolve these discrepancies.

## Resource-sharing between working memory and attention

The time-based resource-sharing model (TBRS; Barrouillet et al., 2004, 2007, 2011) assumes that items are maintained in WM via a “refreshing” process that requires attention, and the same resource that is used to refresh WM is also used for attentional processing of external stimuli. TBRS quantifies the amount of information that can be maintained in WM, as a function of intervening attention or “processing” demands, by varying the time-consumption of processing events during WM maintenance—either by manipulating the number of stimuli to process or the demand level of individual processing tasks. In brief, increasing processing demands impairs WM storage.

While tests of the TBRS model have exclusively examined the impact of attention demands on WM storage (i.e., time-consuming processing limits WM maintenance), studies of WM biasing of selection (e.g., Downing, 2000; Soto et al., 2005; Olivers et al., 2006) have primarily examined the impact of WM content on the allocation of attention (i.e., information maintained in WM determines what gets processed). Together, however, the two literatures suggest that there is a reciprocal trade-off between WM and attention. Indeed, there is a growing consensus that WM can be thought of as attention oriented toward internal representations (Awh and Jonides, 2001; Postle, 2006; Chun, 2011; Gazzaley and Nobre, 2012; Kiyonaga and Egner, 2013). WM for an item can be improved by retrospectively focusing attention on it in response to a cue (i.e., “retro-cue benefit”; Griffin and Nobre, 2003), spatial attention is recruited to maintain WM representations (Awh et al., 1998; Nobre et al., 2004), WM representations can impact behavior just like visually attended stimuli (Kiyonaga and Egner, 2014), and WM and attention have been demonstrated to rely on the same neural mechanisms (Awh et al., 2000; Lepsien et al., 2005; Kuo et al., 2009; Ikkai and Curtis, 2011). Prominent embedded process theories of WM, further, describe activation and maintenance in WM as accomplished by directing an internal focus of attention at long-term memory representations (Cowan, 1988, 2001; Oberauer, 2009; Oberauer and Hein, 2012), and another recent theory (Olivers et al., 2011) suggests that WM content might capture attention only when it is held in that focus of attention.

Accordingly, we have recently proposed that internal (WM) and external (visual selection) prioritization processes share a common attention resource (i.e., the same focus of attention shifts between endogenously and exogenously activated representations), and this resource-sharing—akin to the time-shared refreshing mechanism described by TBRS—might explain why the contents of WM, sometimes unwittingly, impact the allocation of visual attention (Kiyonaga and Egner, 2013). In particular, if the internal maintenance of information occurs via refreshing, and this brief foregrounding places that information in the focus of attention (cf. Johnson et al., 2007; Chun and Johnson, 2011), this will activate the sensory neurons that are responsive to the features of the maintained representations, and sensitivity to matching items in the environment will consequently be increased (even if they are not immediately task-relevant). Here, we adapt the TBRS methodology to test this hypothesis.

As dictated by TBRS, if the focus of attention is continually oriented toward external stimuli for processing, internal refreshing should be hindered, and the magnitude of this hindrance will depend on the relative time-consumption of the external attention demand: more time-intensive demands to direct attention externally will result in less opportunity to refresh the WM content, and worse WM recognition performance. Crucial to our hypothesis, the obstruction of internal refreshing should keep internal content out of the focus of attention, thereby decreasing perceptual sensitivity for stimuli with matching features, which should in turn lead to an attenuated influence of WM content on the selection of external stimuli. In short, if we apply the logic of TBRS more broadly to the relationship between internally geared (e.g., WM content) and externally geared (e.g., visual search) attention, then one can potentially explain (a) why WM content captures attention in the first place (because the two share the same attention resource) and (b) when that capture of attention is likely to occur (when that resource has ample time to switch between internal refreshing and external selection, but not when refreshing is prevented).

## Experiment 1

If our proposal were correct, we should be able to systematically modulate the degree to which internal WM content captures attention by experimentally manipulating the time required for externally-geared attentional processes. To test this possibility, we combined elements of the methods used to test both TBRS and the WM biasing of visual attention. We devised a delayed match-to-sample WM task wherein the time-consumption of delay-spanning attention demands was manipulated by independently varying both the number of intervening visual search processes to be performed, and the amount of available time to perform them (i.e., the rate of presentation). WM items could cue visual search targets or distracters (or neither). We predicted that under a low rate of external attention demands there should be opportunity to regularly refresh the internal representations, which should then influence the allocation of external attention (leading to capture of visual attention by WM content), and be remembered well—regardless of the total number of externally-geared attention processes. Under a high rate of external attention demands, however, refreshing of the internal content should be limited, thus it should be less able to guide external selection (leading to reduced capture of visual attention by WM content), and be remembered more poorly. This pattern of results would support the existence of a common cognitive resource to lie at the root of the relationship between WM representations and visual attention, and validate the applicability of the TBRS approach to study this relationship.

### Methods

#### Participants

Thirty-one volunteers (10 female, median age = 20) gave written informed consent and received course credit or $10.00 payment for their participation. The study was approved by the Duke University Institutional review board.

#### Stimuli and procedure

**Figure 1:**
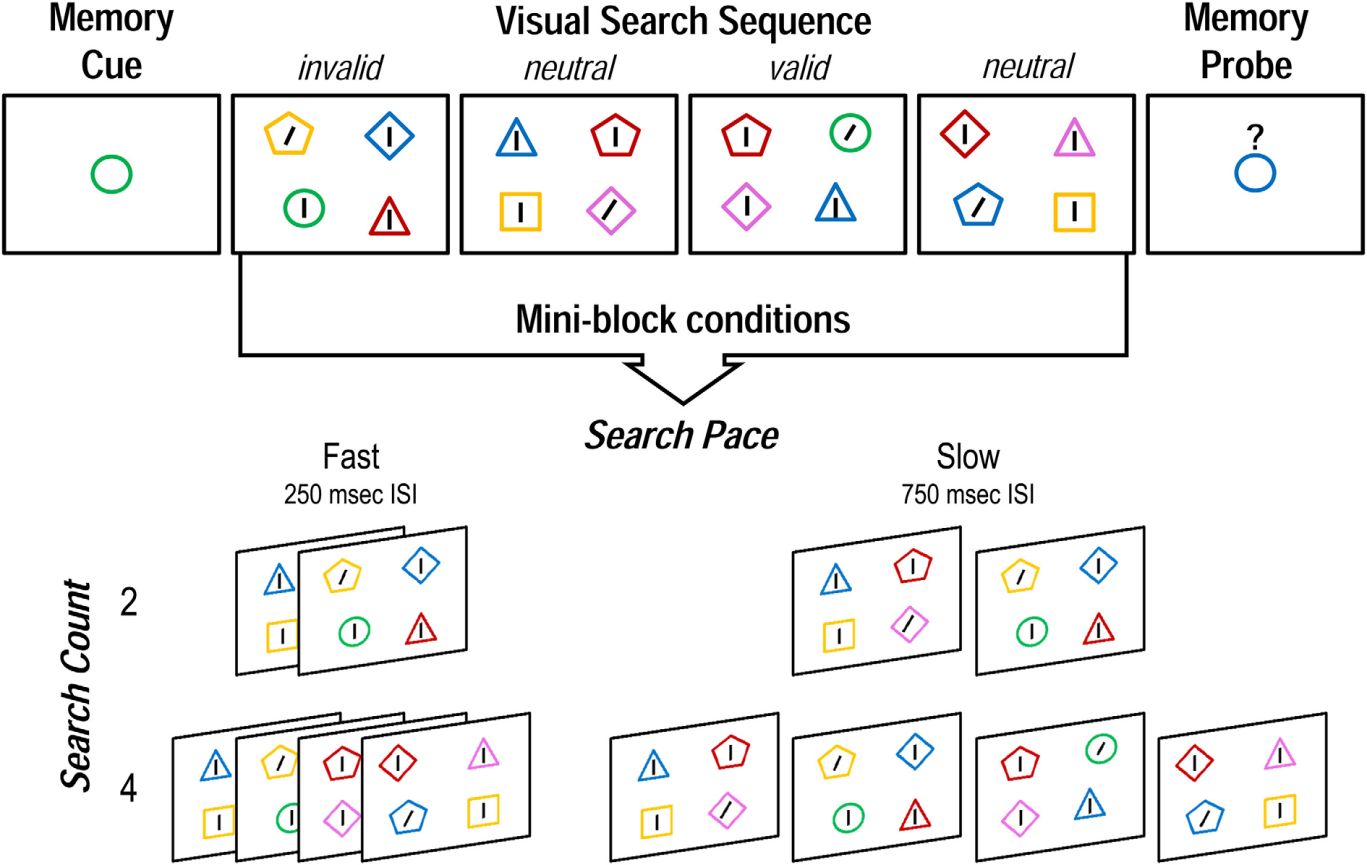
**Structure of a single mini-block, and the four possible conditions of the WM delay-spanning visual search sequence in Experiment 1**. Invalid, neutral, and valid search displays occurred in random order. Experiment 2 was identical except that *search count* varied between 4 and 6, and invalid, neutral, and valid search displays were constrained to occur equally often.

The experiment was programmed and presented using the Psychophysics Toolbox extensions (Brainard, 1997) for Matlab R2010a (Mathworks Inc., Natick, MA, USA) on a Dell Optiplex 960 computer. Stimuli were viewed from approximately 60 cm on an LCD monitor with a 60 Hz refresh rate and a screen resolution of 1280 × 1024 pixels. The task was composed of “mini-blocks,” each comprising a delayed match-to-sample WM test with a series of visual search trials during the delay (wherein WM items could reappear in the search array; cf. Soto et al., 2005). Each mini-block began with the presentation of a central fixation dot for 1500 ms, which was then replaced by a to-be-remembered colored shape cue for 1000 ms. The WM cues were randomly selected from a combination of five shapes (circle, square, triangle, diamond, and pentagon) and five colors (RGB values—red: 255, 0, 0; blue: 0, 0, 255; green: 0, 255, 0; yellow: 255, 255, 0; pink: 255, 0, 255), each subtending a visual angle of approximately 3.8° × 3.8°. The memory cue was then followed by a fixation display of either 250 or 750 ms (depending on condition), then a sequence of either two or four visual search trials, each displayed for 400 ms, and separated by a central fixation cross for either 250 or 750 ms (Figure 1). All stimuli were presented against a gray background (RGB: 128, 128, 128).

Each search display was composed of four lines surrounded by colored shapes—one within each quadrant of the screen at a quasi-random location from trial to trial. Three of the lines were vertical and one—the target—was tilted 25° to the left or right. The participants' task was to indicate the orientation of the one slanted line in each array. The surrounding shapes, each subtending approximately 3.8 × 3.8°, were randomly chosen from the same set as the memory cues, but constrained so that there were no color or shape matches among stimuli in a given search display, and no partial matches to the memory item for that mini-block (though there could be a complete match). Target locations and colored shape stimuli were randomly chosen, thus individual search trials could be valid (memory item reappears surrounding the search target), invalid (memory item reappears surrounding a distracter), or neutral (memory item does not reappear in the search display). After the visual search sequence, a single colored shape memory probe appeared at the center of the screen underneath a question mark for 1500 ms. The participants' task was to indicate whether this shape was the same or different from the original memory cue. The memory probe was always a whole or partial match to the memory cue (never different on both color and shape), and match and non-match probes occurred equally often and in random order.

The number of visual search trials between memory cue and probe (*search count*: two vs. four), and the duration of the inter-stimulus interval between search arrays (*search pace*: 250 ms fast vs. 750 ms slow) were combined to produce four different mini-block conditions, (total search sequence durations: *two fast*, 1.55 s; *two slow*, 3.05 s; *four fast*, 2.85 s; *four slow*, 5.35 s), which were presented in random order. This allowed us to look at the unique contributions to later memory of both the number of operations that needed to be performed during the WM delay, and the pace at which they occurred. Since the WM cue could be a valid, invalid, or neutral indicator of the search target, we were able to evaluate the impact of mini-block condition on the effect of WM cueing validity. After a practice block, participants completed eight experimental runs, each consisting of 24 mini-blocks (i.e., 48 mini-blocks per condition). The proportion of valid, invalid and neutral trials was unconstrained within a mini-block, but any given trial type occurred equally often across each mini-block condition. Across the entire experiment, one in three search trials were neutral (32 per *two fast* and *two slow*, 64 per *four fast* and *four slow*); of the remaining two thirds when the memory item reappeared in the search, it corresponded to a distractor three out of four times (invalid: 48 per *two fast* and *two slow*, 96 per *four fast* and *four slow*). Thus, one in six total search trials were valid (16 per *two fast* and *two slow*, 32 per *four fast* and *four slow*). We predicted that, if internal representations guide external selection when they are being refreshed, a fast search pace would dampen the influence of cue validity, and impair memory recognition, irrespective of search count.

### Results and discussion

**Figure 2:**
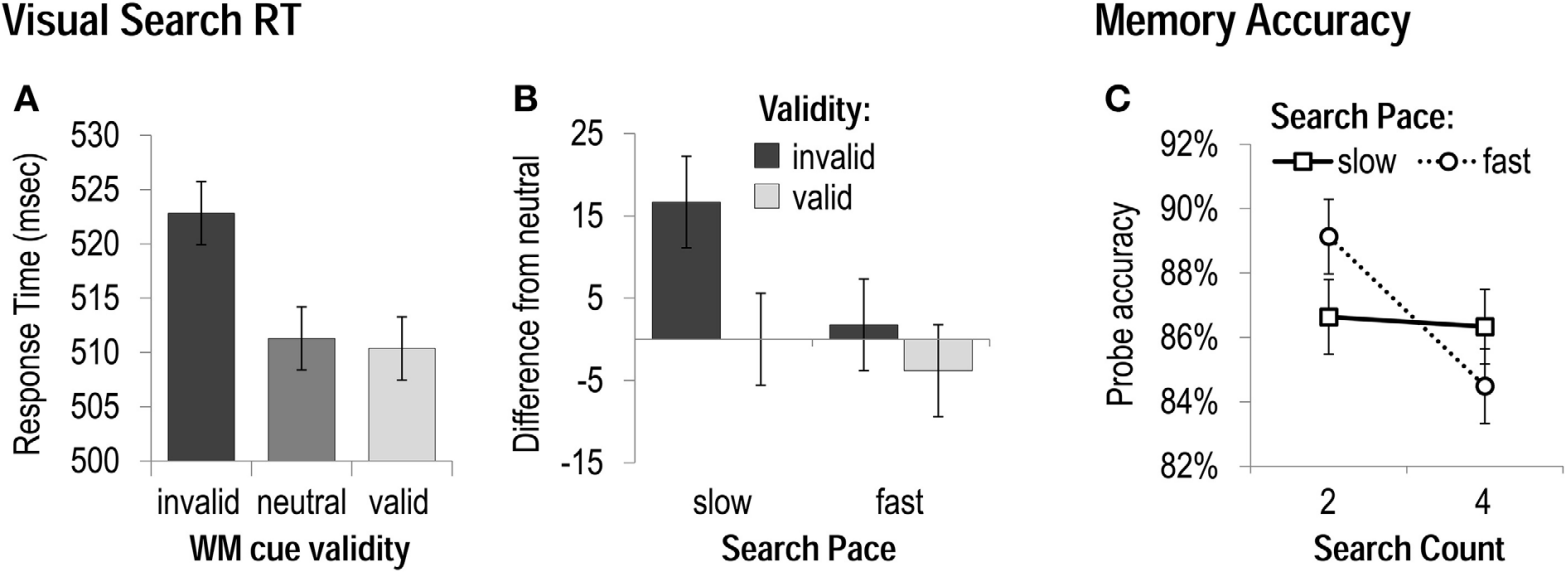
**Task performance in Experiment 1**. **(A)** Response Times—collapsed across all mini-block conditions—when WM cues were matching visual search distracters (invalid), targets (valid), or did not reappear in the search display (neutral). **(B)** The magnitude of costs (slowing by invalid cues) and benefits (speeding by valid cues) to visual search as a function of the rate of search presentation. **(C)** Accuracy on memory probes for each mini-block condition.

We performed 3 × 2 × 2 ANOVAs including the factors of *WM cue validity* (invalid, neutral, valid), *search count* (two vs. four), and *search pace* (fast vs. slow), on visual search performance measures. Measures included accuracy (% correct) and mean correct response time (RT; within 3 *SD* of an individual's mean) for trials when the memory probe was also correctly performed. Visual search accuracy was unaffected by WM-matching or mini-block condition, while RT was sensitive to WM cue validity, *F*_(1, 29)_ = 6.83, *p* < 0.05, and search pace, *F*_(1, 29)_ = 19.65, *p* < 0.001, though not to search count, *p* > 0.2. Participants were significantly slower to correctly discriminate the search target when a WM matching item appeared as a distracter (invalid) than when the memory item coincided with a target (valid) or did not reappear at all (neutral; Figure 2A). While participants were faster overall during the fast-paced conditions, this factor of pace also interacted with WM cue validity, *F*_(1, 29)_ = 4.43, *p* < 0.05. Most importantly to our research question, the magnitude of the RT cost of an invalid visual search distracter (invalid RT-neutral RT) that was robust during slow-paced conditions, *t*_(30)_ = 4.3, *p* < 0.001 was eliminated when the search trials had to be performed at a fast pace, *t*_(30)_ = 0.8, *p* > 0.4 (Figure 2B).

We also performed 2 × 2 ANOVAs including the factors of *search count* (two vs. four), and *search pace* (fast vs. slow) on WM probe accuracy and RT. Accuracy on the delayed-recognition memory test was better when it was only preceded by two search trials (vs. four), *F*_(1, 30)_ = 4.46, *p* < 0.05. While there was no main effect of search pace on probe accuracy, *p* > 0.8, the search pace and count did interact, *F*_(1, 30)_ = 6.44, *p* < 0.05 (Figure 2C). Participants were equally accurate in the slow-paced conditions, whether they had performed two or four search trials, *p* > 0.9; in the fast-paced conditions, however, they were less accurate after four search trials (vs. two), *t*_(30)_ = 3.32, *p* < 0.01. Memory probe accuracy was best overall during the *two fast* condition (which also resulted in the shortest delay between memory cue and probe), and worst during the *four fast* condition (even though this condition had a shorter delay between cue and probe than the *four slow* condition). Participants were also faster to correctly respond to the memory probe when they only had to complete two search trials (vs. four), *F*_(1, 30)_ = 22.14, *p* < 0.001, and faster when they had to be completed at a fast pace (vs. slow), *F*_(1, 30)_ = 4.29, *p* < 0.05, but there was no interaction between these factors, *p* > 0.4.

In support of the notion that WM content may capture visual attention when it is being rehearsed or refreshed, the slowing of visual search by a WM matching distracter was curtailed when the WM delay was filled with a series of fast-paced visual search trials—which would presumably occupy attention externally and hinder refreshing of internal WM content. This effect of validity on search speed was not sensitive to the total number of search trials that needed to be completed—only to the pace of their completion. In support of the interpretation that this attenuation of attentional capture is due to the blockage of refreshing, WM accuracy was also worst after performing four visual search trials at a fast pace. This memory impairment cannot be explained by just the time-related decay of WM representations, since probe accuracy was better after four search trials performed at a slow pace, which yielded a longer delay between WM cue and probe. This is consistent with the idea that the fast-paced visual search sequence monopolized attention resources, leaving little time to direct attention toward refreshing the WM content. As a consequence, the to-be-remembered items were less able to capture visual attention in the search.

We did, however, observe two unexpected results. First, there was no overall benefit to visual search of a memory-matching target. Our ability to detect a validity benefit may have been undermined by the small proportion (and total number) of these valid trials, which constituted only one sixth of all trials. Second, memory probe performance was best after two fast-paced visual search trials, counter to our expectation that the fast search pace would impair memory performance, regardless of the number of operations to be performed (cf. Barrouillet et al., 2011). However, this finding could be attributable to the fact that the delay between WM cue and probe was so short in this condition (1.55 s) that it may have been possible to maintain and identify the probe without needing to direct attention toward refreshing the WM representation. We addressed these unexpected results, and sought to confirm our interpretation of them, in Experiment 2.

## Experiment 2

The conclusion that WM refreshing and visual search share a resource would be strengthened if we were also able to demonstrate that (1) the visual search *benefits* of a WM-matching target are attenuated when WM refreshing is hampered, and (2) fast-paced external attention demands can interfere with memory accuracy, regardless of the number of search operations to be performed. In Experiment 2, thus, we (1) altered the proportion of valid, invalid, and neutral visual search trials to increase the incidence of valid trials, and (2) increased the number of search trials to be performed in all mini-blocks, to prolong the minimum delay between WM cue and probe so that maintenance of the WM representation would require refreshing.

### Methods

#### Participants

Twenty volunteers (13 female, median age = 18.5) gave written informed consent and received course credit or $10.00 payment for their participation. The study was approved by the Duke University Institutional review board.

#### Stimuli and procedure

The experimental paradigm was identical to Experiment 1 with the exception of two important changes: (a) The proportion of valid, invalid, and neutral visual search trials was constrained so that they each occurred equally often (⅓ of all trials) and (b) the minimum number of visual search operations to perform was increased from two to four (thus raising the minimum time interval from WM cue to probe from 1.55 to 2.85 s), and the maximum number from four to six. In Experiment 2, consequently, participants completed either four or six search trials, at either a slow or fast pace, during each WM delay. Again, these were combined to produce four mini-block conditions (total search sequence durations: *four fast*, 2.85 s; *four slow*, 5.35 s; *six fast*, 4.15 s; *six slow*, 7.65 s), which were presented in random order. The participant's task was identical to that in Experiment 1: to indicate the orientation of the slanted line target in every visual search array, and to indicate whether a probe was a match or non-match to the WM cue presented prior to the visual search sequence. After a practice block, participants completed 6 experimental runs, each consisting of 24 mini-blocks (i.e., 36 mini-blocks per condition; 48 search trials of each validity type per *four fast* and *four slow*, 72 search trials of each validity type per *six fast* and *six slow*).

### Results and discussion

**Figure 3:**
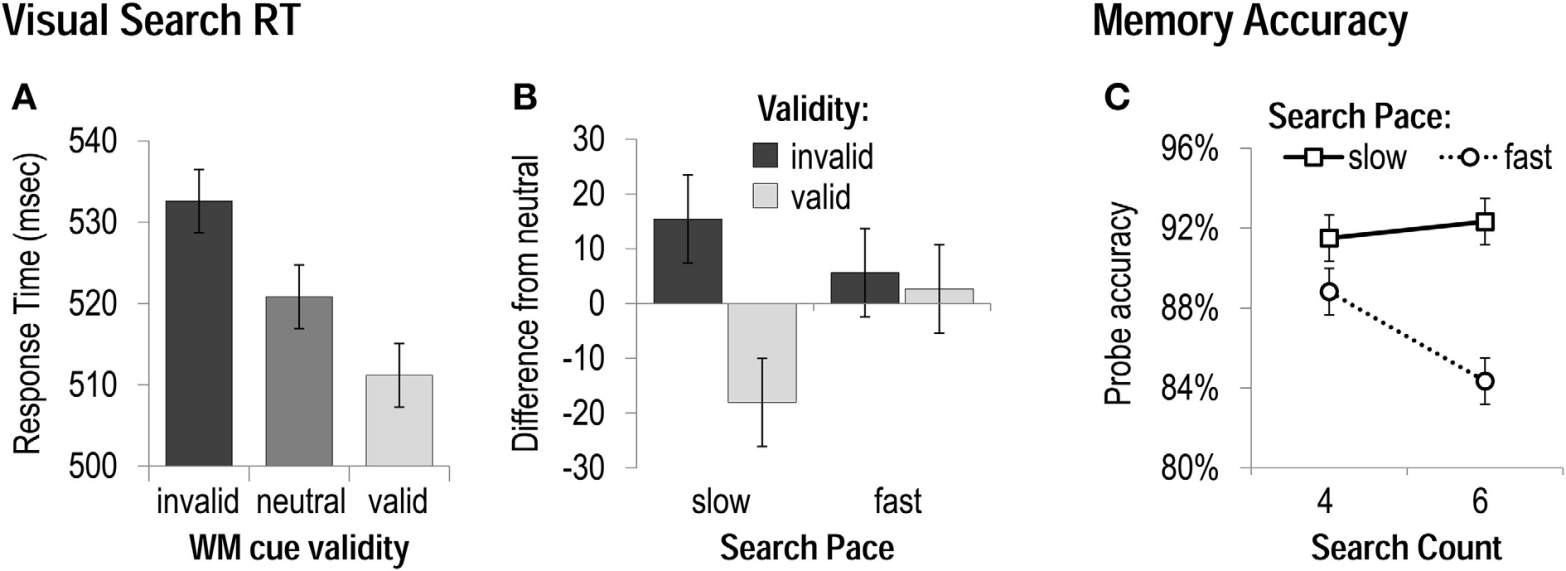
**Task performance in Experiment 2**. **(A)** Response Times—collapsed across all mini-block conditions—when WM cues were matching visual search distracters (invalid), targets (valid), or did not reappear in the search display (neutral). **(B)** The magnitude of costs (slowing by invalid cues) and benefits (speeding by valid cues) to visual search as a function of the rate of search presentation. **(C)** Accuracy on memory probes for each mini-block condition.

As in Experiment 1, we performed 3 × 2 × 2 ANOVAs including the factors of *WM cue validity* (invalid, neutral, valid), *search count* (four vs. six), and *search pace* (fast vs. slow), on visual search accuracy and RT. Accuracy was best during slow-paced mini-blocks, *F*_(1, 19)_ = 28.05, *p* < 0.001, and best with valid cueing (and worst with invalid WM cueing), *F*_(1, 19)_ = 4.41, *p* < 0.05, and there was no effect of search count, nor any significant interactions between factors. Visual search RT was sensitive to both WM-matching and mini-block condition. Search performance was fastest with valid cueing, and slowest with invalid cueing, *F*_(1, 19)_ = 7.67, *p* < 0.01 (Figure 3A). Relative to the neutral condition, search was marginally slowed by a WM-matching distractor, *t*_(19)_ = 2.0, *p* < 0.06, and, critical to the goals of Experiment 2, expedited by a WM-matching target, *t*_(19)_ = 2.6, *p* < 0.05. Search time was slightly slower when six search trials had to be completed (vs. four), *F*_(1, 19)_ = 4.53, *p* < 0.05, and faster during the fast paced conditions (vs. slow), *F*_(1, 19)_ = 14.59, *p* < 0.001. Search count interacted with pace, *F*_(1,19)_ = 6.15, *p* < 0.05, in that the speed difference between four and six trial sequences was only evident in the slow-paced condition. Search count also interacted with WM cue validity, *F*_(2,38)_ = 5.29, *p* < 0.01, in that the magnitude of the effect of validity was larger when there were fewer search trials to be performed. As observed in Experiment 1, the effect of WM validity also interacted with search pace, *F*_(2,38)_ = 3.71, *p* < 0.05. Most importantly to the goals of Experiment 2, both the cost of a WM-matching distractor (invalid RT-neutral RT), *t*_(19)_ = 1.5, *p* > 0.1, and the benefit of a WM-matching target (valid RT-neutral RT), *t*_(19)_ = 0.5, *p* > 0.5, were eliminated during the fast pace search sequences (Figure 3B).

We also performed 2 × 2 ANOVAs including the factors of *search count* (two vs. four), and *search pace* (fast vs. slow), on WM probe accuracy and RT. Memory recognition speed was unaffected by mini-block condition. Unlike Experiment 1, memory accuracy was not significantly influenced by the number of searches that had been performed, but it was worse after fast-paced visual search sequences of either four or six trials, *F*_(1, 19)_ = 12.41, *p* < 0.01 (Figure 3C). This effect of visual search pace on memory performance did interact, however, with the search count, *F*_(1, 19)_ = 9.64, *p* < 0.01, in that the fast pace had a greater disruptive effect when a greater number of search trials had been completed. Thus, although TBRS would predict WM performance to be unaffected by the total number of processing events (as long as they occur at a constant pace), in the current task context, WM storage appears to be influenced by a combination of the absolute amount of processing that occurs between memory cue and probe (cf. Ricker and Cowan, 2010), as well as the amount of available time during the delay to refresh the WM content. One possibility is that the processing manipulation used here prevented WM refreshing to the point that the time-related decay proposed by TBRS (when items cannot be refreshed) had an accumulating impact on WM as more time elapsed (i.e., when six searches had to be performed rather than four). Alternatively, given that memory items could be fairly easily verbalized, perhaps a verbal rehearsal strategy was used to provide some degree of protection against the fast-paced search sequence at shorter delays (cf. Vergauwe et al., 2014).

In sum, both slowing of visual search by a WM-matching distracter and speeding by a WM-matching target were dampened when the WM delay-spanning search sequence was presented at a fast pace, and presumably prevented the WM content from being refreshed. Consequently, memory probe accuracy was also worse during these fast-paced, refreshing-preventing conditions. Thus, regardless of the number of operations to be performed during a WM delay, or the total duration of that delay, biasing of visual search was minimized when the delay was filled with an attention-demanding visual task that left little time for refreshing of WM content.

## General discussion

### Summary

In two experiments, we observed that high frequency external stimulus processing demands can curb the extent to which visual selection favors items in the environment that match the contents of WM. Akin to the manner in which heightened perceptual load can limit the processing of perceptual distracters (Rees et al., 1997), here an increased external load modulated both the influence and retention of internal content. These data provide further evidence of a shared resource between internal (i.e., WM maintenance) and external (i.e., visual attention) selection processes, they implicate that resource as a source of visual capture by WM contents, and they likewise illuminate why WM contents might capture visual attention in some situations but not others. The results are consistent with earlier observations that time demands and cognitive load can limit the impact of WM on visual attention (Soto and Humphreys, 2008; Dombrowe et al., 2010; Dalvit and Eimer, 2011), and in keeping with the broader notion that WM is akin to internally-oriented attention (Postle, 2006; Chun, 2011; Gazzaley and Nobre, 2012; Kiyonaga and Egner, 2013). The TBRS approach—of manipulating processing demand to measure its impact on WM storage capacity—may thus offer a promising means of investigating the related phenomenon of WM biasing of attention.

### Alternative interpretations

Recent studies suggest that the delay following presentation of a WM cue may be used to consolidate the WM representation, and that different consolidation times can lead to different rates of forgetting (Ricker and Cowan, 2014; Vergauwe et al., 2014). Given that the duration of the interval between the WM cue and visual search sequence varied with trial condition in the current study, it may be possible that the fast-paced conditions provided less time to consolidate the WM item, which may have then had less impact on search performance. We point out, however, that we report visual search performance only for trials with a correct response on the memory probe; so, it is unlikely to be the case that WM cues failed to impact search simply because they were never consolidated/remembered in the first place. On average, memory probe performance was worst after fast-paced visual search sequences (but still well above chance), suggesting that our manipulation was effective at limiting WM refreshing. That overall impediment to refreshing also manifested itself as a failure for the WM content to capture visual attention under those fast-paced conditions, even when the WM content was ultimately correctly recalled. It is possible that when refreshing was impeded—but items still correctly remembered—WM representations were designated a different “accessory” status outside the focus of attention (cf. LaRocque et al., 2014), whereby they would be less likely to impact visual selection (cf. Olivers et al., 2011), but were able to be later reinstated into the focus of attention for WM retrieval (Kiyonaga et al., 2012).

A number of other alternatives—besides a simple refreshing impediment—might explain the increased loss of information from WM when more visual search trials were given at a faster pace. It could be argued, for instance, that the visual search manipulation used here does not place demands exclusively on visual attention *per se*, but also response selection and execution mechanisms that could interfere with WM maintenance more than the visual demands. Additionally, the possibility that directing attention to objects in visual search leads to their transfer into WM (Schmidt et al., 2002), or that tracking previously attended locations in visual search taxes WM (Castel et al., 2003), might both interfere with concurrent WM representations. The serial order in a box model (SOB; e.g., Oberauer and Lewandowsky, 2008; Lewandowsky et al., 2009; Oberauer et al., 2012), likewise, attributes the decay of WM to interference from distractors, which require time to be removed from the limited-capacity store. Considering our results within the SOB framework, we presume that the attention resource that is required to remove interference from WM is the same one that would otherwise be directed at maintaining the WM content; thus, the time-consumption of resolving interference would also dampen the biasing of visual attention toward WM-matching items. We would, therefore, argue that although by different means, each of these alternatives prevents a limited shared attention resource from being oriented toward the to-be-remembered content. That is, regardless of the specific barrier to directing attention at WM content, when such a barrier exists, that internal content will be prevented from guiding visual selection. A potentially informative means to further test the refreshing mechanism proposed herein would be to examine the magnitude of WM biasing across the delay period (i.e., on the first search display vs. the last in the sequence), in a task with greater trial numbers of each condition, to illuminate how the WM influence changes as time and processing demands accrue.

### Implications

Competition between the domains of WM and attention has typically been revealed by maximizing demands to the point that the presumed shared resource is fully occupied, and processing in one or both domains consequently suffers (e.g., De Fockert, 2001; Barrouillet et al., 2004). Accordingly, we found that WM accuracy was impaired when the shared resource was tied up by requirements to direct attention externally. Within the unique task structure employed here, however, it was in the lower visual attention demand conditions—because the shared resource was available to refresh WM representations—that internal content was able to impact external attentional selection. This study thus marks a novel methodological approach to understanding the link between WM and visual selection. By applying the TBRS structure to the dual-task WM-visual search paradigm, we have found further evidence that the contents of both WM and attention are determined by a shared selection mechanism that alternates between internal and external domains, and is limited in the amount of information it can process in a given period of time. Most importantly, when this selection resource has ample time to regularly refresh internal WM content, that content will influence external selection, but when such refreshing is hindered external selection will be unaffected by internal WM representations.

While the findings reported here indicate that the biasing of visual attention by WM contents only occurs when task constraints allow refreshing of WM representations, however, it is not the case that such WM biasing is only prevented when external task demands are maximal; it has in fact been shown that external search targets can be effectively prioritized even in the absence of stringent time demands. Several studies, for instance, have failed to find an impact of WM content on visual attention when the visual search target changes from trial to trial (Downing and Dodds, 2004; Houtkamp and Roelfsema, 2006; Olivers, 2009; Peters et al., 2009). It could be the case that when the search target is variable, it is more demanding to maintain, and thus remains in the focus of attention where it will guide visual search above and beyond other internal material (cf. Woodman et al., 2007). When the WM content is known to be predictably harmful to the current task (Woodman and Luck, 2007; Carlisle and Woodman, 2011; Kiyonaga et al., 2012), top-down strategies might be applied to reallocate the WM content to an accessory status outside the focus of attention, and thereby limit its influence on visual attention. Other studies have varied the perceptual difficulty of the visual search (Han and Kim, 2009) or the time window for responding to the search (Dalvit and Eimer, 2011), and have explained the subsequent attenuation of WM biasing as a result of increased cognitive control under these conditions, but both findings are highly consistent with the premise of the present study: that maintenance of internal WM content and demands on externally-geared attention rely on a shared, limited resource, and WM content will not bias visual attention when that shared resource is not regularly directed toward refreshing the WM content in the focus of attention. Time-consuming external attention demands are one way of limiting WM refreshing, but any task attribute that limits the extent to which attention is directed at the WM content should interfere with both WM recall (though sometimes impacting speed or precision rather than accuracy) and WM biasing of selection of external stimuli. The current data thus demonstrate how and when internal representations capture attention, although the specific task setup reflects only one of a number of ways to probe the boundary conditions of this interaction.

The limits of this shared prioritization resource can furthermore be applied to explain a number of other observations that have been interpreted as indicating that WM and attention demands are discrete (e.g., Woodman et al., 2001; Hollingworth and Maxcey-Richard, 2012; Hollingworth and Hwang, 2013; Rerko et al., 2014). Specifically, the absence of interference between unrelated WM and attention demands would be expected if the shared selection mechanism was given enough time to comfortably alternate between tasks. One study, for instance, required participants to complete a visual search during the delay interval of a change detection task, and concluded that sustained attention was not required for selective maintenance in WM (Hollingworth and Maxcey-Richard, 2012). In this example, participants were given 2000 ms to complete the visual search, but they typically took less than half of that time to respond, indicating that there was ample time within the visual search window to redirect the focus of attention back to refresh the WM content. Absolutely unbroken sustained attention is therefore unnecessary to maintain WM, as long as there is some opportunity to occasionally refresh the WM content. The mechanism tested herein can thus be used to reconcile a collection of seemingly inconsistent findings.

## Conclusions

A complete understanding of the prioritization between internal and external domains will require future examination of whether the boundaries of this mechanism are, for instance, in the rate of decay of internal representations (in the absence of refreshing), the efficiency of assimilating external information, or refreshing internal information (cf. Barrouillet and Camos, 2012), the speed of alternating between processes and domains, or the volume of the resource pool. If what have previously been considered distinct cognitive concepts (i.e., WM and attention) rely on a common resource, and that resource can be trained or enhanced (e.g., Anguera et al., 2013; Kundu et al., 2013), then a specific characterization of its underlying capacity can contribute to understanding and enhancement of information processing in multiple realms of cognition. These data suggest not only that WM and external attention are related, but that they are reciprocal because they both hinge on a common attention resource. Although the notion of reciprocity between WM and attention is not a brand new one, the bridging of methods previously employed to study distinct cognitive operations represents a new framework within which to study and understand the means by which we select and process information in all domains.

### Conflict of interest statement

The authors declare that the research was conducted in the absence of any commercial or financial relationships that could be construed as a potential conflict of interest.
